# Invasive Complications and Infection-Related Reconstruction in Bicuspid Versus Tricuspid Native Aortic Valve Endocarditis: A 12-Year Surgical Cohort Study

**DOI:** 10.3390/jcdd13100521

**Published:** 2026-10-16

**Authors:** Yuanhang Zhai, Lanlin Zhang, Shuofang Ren, Xinpei Liu, Yuheng Dong, Guotao Ma, Shangdong Xu, Jun Zheng

**Affiliations:** Department of Cardiac Surgery, Peking Union Medical College Hospital, Chinese Academy of Medical Sciences & Peking Union Medical College, No. 1 Shuai Fu Yuan, Dongcheng District, Beijing 100730, China

**Keywords:** bicuspid aortic valve, infective endocarditis, invasive endocarditis, paravalvular complication, reconstruction

## Abstract

Background: Detailed operative evidence comparing invasive anatomy and reconstruction between bicuspid aortic valve (BAV) and tricuspid aortic valve (TAV) cases of native-valve infective endocarditis is limited. We examined the association of BAV morphology with non-leaflet invasive complications and infection-related reconstruction. Methods: We retrospectively studied 317 adults who underwent surgery for native aortic valve infective endocarditis from 2013 through 2025 (113 BAV; 204 TAV). The primary outcome was any non-leaflet invasive complication; infection-related reconstruction was a secondary operative outcome reflecting operative management of invasive anatomy. Modified Poisson regression estimated adjusted prevalence ratios (aPRs), corresponding 95% confidence intervals (CIs), and marginally standardized absolute differences. Site-specific and invasive-subgroup analyses were exploratory. Results: Patients with BAV were younger, more frequently male, and had a lower comorbidity burden. Non-leaflet invasive complications occurred in 44.2% of patients with BAV and 18.6% with TAV (aPR, 2.08; 95% CI, 1.41–3.06; adjusted absolute difference, 21.1 percentage points; 95% CI, 9.6–32.6). Abscess involving non-leaflet structures was more frequent in BAV than in TAV (43.4% vs. 16.7%; *p* < 0.001). In the full cohort, aortic annular and left ventricular outflow tract involvement were more frequent in BAV. Infection-related reconstruction was performed in 31.9% of patients with BAV and 15.2% of those with TAV. Within the invasive subgroup, reconstruction was performed in 72.0% and 81.6% of BAV and TAV cases, respectively (unadjusted PR, 0.88; 95% CI, 0.70–1.11). Early mortality was low in both groups, and the adjusted analysis did not identify a clear difference in long-term mortality. Conclusions: Bicuspid morphology was associated with a higher prevalence of non-leaflet invasive complications, predominantly abscess, and a higher overall frequency of infection-related reconstruction.

## 1. Introduction

Infective endocarditis (IE) remains a life-threatening disease despite advances in diagnosis, antimicrobial therapy, and surgery. Aortic valve IE (AV-IE) is especially challenging when infection extends beyond the leaflets, because radical debridement may leave defects involving the annulus, aortic root, left ventricular outflow tract (LVOT), or adjacent chambers. Such extension can necessitate reconstruction in addition to valve repair or replacement and is central to operative planning and prognosis [1,2].

Bicuspid aortic valve (BAV) is the most common congenital cardiac valvular anomaly and carries a substantially increased lifetime risk of native-valve IE [3,4]. Across observational cohorts, patients with BAV-associated IE have consistently been younger and predominantly male, with a higher prevalence of periannular or other intracardiac complications than patients with tricuspid aortic valve (TAV) IE [5,6,7,8,9,10,11]. Contemporary surgical and matched-cohort studies have further characterized operative burden and survival, but the adjusted association between valve morphology and long-term outcomes remains uncertain [12,13,14].

Most previous comparisons have treated periannular abscess or intracardiac complications as broad outcomes. Detailed operative data on the individual non-leaflet structures involved and the reconstructive procedures performed after debridement remain limited. Defining these anatomical and operative relationships may improve characterization of the surgical phenotype without implying a distinct route of infection spread or a morphology-specific operative strategy.

Building on the established association between BAV and periannular complications, this study provides a standardized operative characterization of the non-leaflet structures involved and the infection-related reconstructions performed, with exploratory comparisons among patients with established invasion. The adjusted analysis focused on any non-leaflet invasive complication as the single primary outcome. Infection-related reconstruction was evaluated as a secondary operative outcome in the full cohort and among patients with established invasion. Early postoperative outcomes and long-term survival were evaluated as secondary clinical context.

## 2. Materials and Methods

### 2.1. Study Population

**Figure 1:**
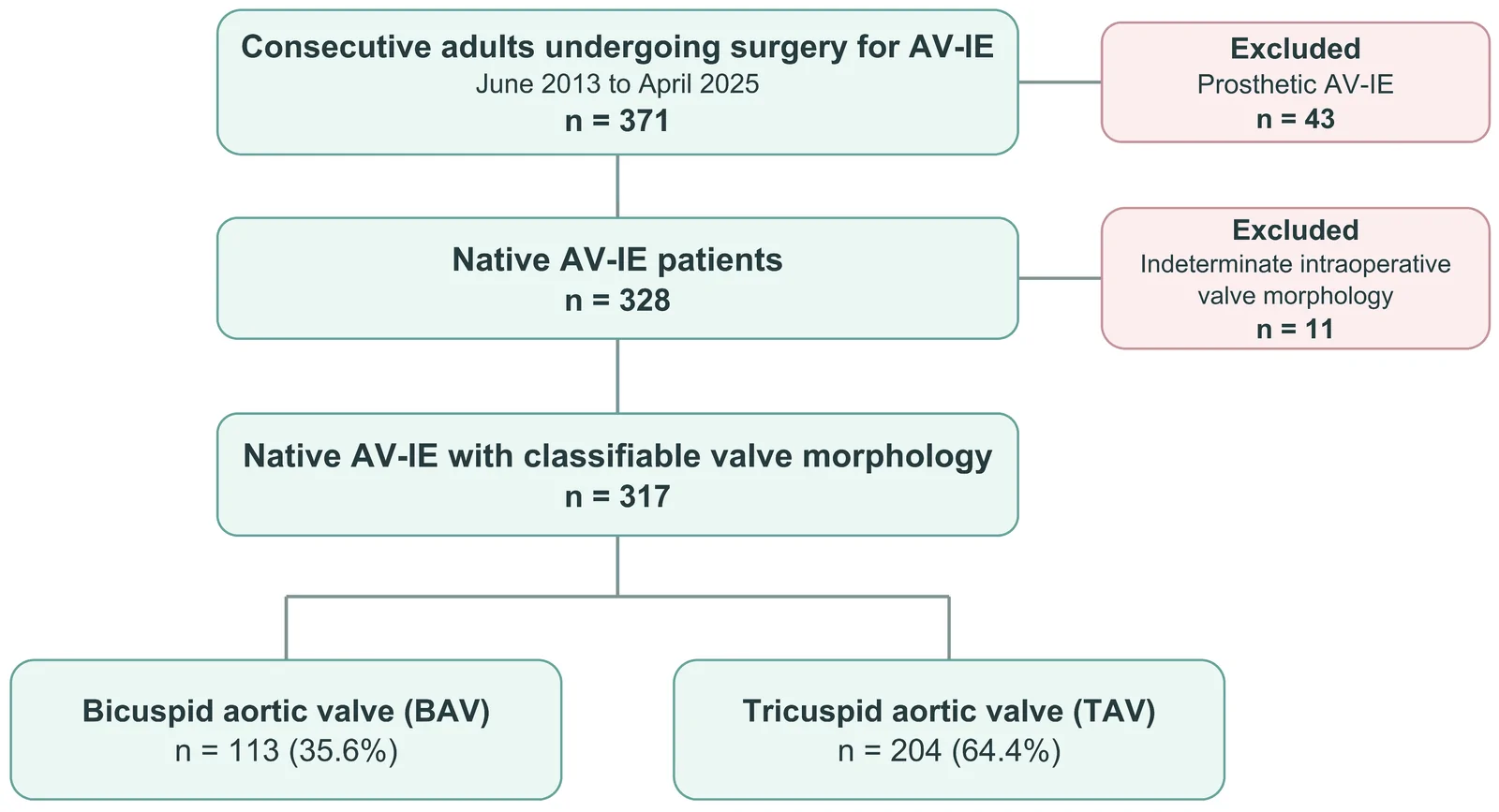
Study Flow Diagram and Cohort Selection. AV-IE, aortic valve infective endocarditis; BAV, bicuspid aortic valve; TAV, tricuspid aortic valve.

This retrospective, single-center study screened consecutive adults who underwent cardiac surgery for AV-IE at Peking Union Medical College Hospital between June 2013 and April 2025. IE was diagnosed according to the modified Duke criteria [15]. Surgical indications were assessed according to the European Society of Cardiology guidelines applicable at the time of treatment. Patients with prosthetic aortic valve endocarditis or indeterminate native aortic valve morphology were excluded. The analysis was restricted to patients whose native valve morphology could be reliably classified as BAV or TAV. No unicuspid or quadricuspid aortic valves were identified among patients with classifiable native valve morphology (Figure 1).

The Ethics Review Committee of Peking Union Medical College Hospital approved the study (I-26PJ0479) and waived individual informed consent because of the retrospective design. Data used for analysis were deidentified.

### 2.2. Data Collection and Definitions

Demographic, clinical, laboratory, microbiological, echocardiographic, operative, postoperative, and follow-up data were obtained from the institutional database and electronic medical records. Original operative reports were available for all 317 included patients and assessed using a prespecified checklist covering valve morphology, non-leaflet invasive complications, anatomical sites, and infection-related reconstruction, with no missing data for these operative variables.

Two investigators independently reviewed the original operative reports and classified valve morphology, non-leaflet invasive complications, and infection-related reconstruction according to prespecified definitions. Before adjudication, inter-reviewer reliability was assessed using percentage agreement and Cohen’s κ; disagreements were resolved through joint review with a third investigator. Valve morphology was classified primarily from operative findings, supported by preoperative echocardiography; when discordant, operative findings took precedence if leaflet configuration was reliably identifiable before or during debridement. Concomitant congenital heart disease was defined as congenital structural cardiac abnormalities other than bicuspid aortic valve morphology. The interval from the first documented diagnosis of IE to surgery was categorized as <24 h, 24 h to 5 days, or >5 days.

A leaflet abscess was confined to aortic leaflet tissue. An abscess involving non-leaflet structures involved at least 1 structure beyond the leaflets, irrespective of a coexisting leaflet abscess or the inferred direction of spread. The primary anatomical outcome, any non-leaflet invasive complication, comprised an abscess involving non-leaflet structures, pseudoaneurysm, or intracardiac fistula. Non-leaflet invasive complications were classified based on documented intraoperative findings, with preoperative transthoracic echocardiography (TTE), transesophageal echocardiography, cardiac computed tomography, and histopathology serving as supplementary evidence when available. Components could overlap.

Anatomic structures involved by non-leaflet invasive complications included the aortic annulus, aortic root wall or sinus, mitral-aortic intervalvular fibrosa, basal interventricular septum, ventricular surface of the anterior mitral leaflet, left ventricular free wall, left atrial roof, right atrium, right ventricle or right ventricular outflow tract, and other adjacent structures. Right-sided involvement was recorded only when operative review documented direct extension from AV-IE rather than independent right-sided valve infection. LVOT involvement was an inclusive descriptor and was not counted as an additional structure. Multiregional involvement was defined as involvement of at least 2 of the listed structures.

The secondary operative outcome, any infection-related reconstruction, comprised annular reconstruction, aortic sinus patch reconstruction, infection-related root replacement, mitral-aortic intervalvular fibrosa reconstruction, interventricular septal reconstruction, fistula repair, or reconstruction of another adjacent structure after debridement. Mitral-aortic intervalvular fibrosa reconstruction included localized patch reconstruction and the Commando and Hemi-Commando procedures. Procedures could overlap, but each patient was counted once in the composite outcome.

Infection-related root replacement was defined as replacement required for infection-related tissue destruction that precluded root preservation or patch reconstruction. Root replacement solely for preexisting aortopathy or dilatation was excluded. The non-age Charlson comorbidity score was calculated without the age component [16].

Reconstruction of infection-related structural defects was performed using bovine pericardium or autologous pericardium. Aortic root replacement was performed using a Bentall procedure with either a mechanical or bioprosthetic valved conduit.

### 2.3. Outcomes and Follow-Up

The primary outcome was any non-leaflet invasive complication. Infection-related reconstruction was evaluated as a secondary operative outcome reflecting the surgical response to invasive anatomy. Its overall frequency and component procedures were described in the full cohort and among patients with established invasion. Individual anatomical sites, multiregional involvement, and reconstruction conditional on established invasion were exploratory. Early outcomes included major postoperative complications, intensive care unit and postoperative hospital length of stay, in-hospital mortality, and 30-day mortality.

Follow-up was conducted through scheduled outpatient visits and telephone interviews, with events verified from medical records and death certificates when available, through 24 August 2026. Long-term survival was measured from surgery to all-cause death or the last date confirmed alive. Three patients lost to follow-up were censored at their respective last-known-alive dates.

### 2.4. Statistical Analysis

Continuous variables were summarized as mean (standard deviation) or median (interquartile range), as appropriate, and categorical variables as number (percentage). BAV and TAV groups were compared using the 2-sample *t* test or Wilcoxon rank-sum test for continuous variables and Pearson chi-square or Fisher exact test for categorical variables. Absolute standardized mean differences (SMDs) quantified baseline differences.

The association between BAV morphology and the primary outcome was evaluated using modified Poisson regression with HC0 robust sandwich variance and TAV as the reference [17]. A secondary model using the same covariates summarized the overall association with infection-related reconstruction and was interpreted descriptively because reconstruction reflected both invasive anatomy and surgical decision-making. Prespecified covariates were age, sex, surgical year, and the non-age Charlson comorbidity score. These variables were selected to standardize the associations across the cohort distributions of demographic characteristics, baseline comorbidity burden, and calendar period. Variables reflecting infection presentation, anatomical extent, or operative management were not included because they could lie on the pathway between valve morphology and the study outcomes or overlap with the outcomes themselves. Age and surgical year were modeled per 10- and 5-year increases, respectively. Adjusted prevalence ratios (aPRs) with 95% confidence intervals (CIs) were reported. Adjusted absolute differences were estimated by marginal standardization and expressed in percentage points; CIs were calculated with the delta method and robust covariance matrix. Both models included all 317 patients, and no imputation was performed. Estimates were interpreted as adjusted associations rather than independent causal effects.

Sensitivity analyses separately excluded patients with concomitant mitral valve IE and patients with concomitant congenital structural heart disease other than BAV morphology itself and used abscess involving non-leaflet structures as a narrower anatomical outcome. Functional forms in the primary and secondary models were assessed with restricted cubic splines using 3 and 4 knots for age and 3 knots for surgical year. Individual anatomical and reconstructive comparisons, including analyses restricted to patients with non-leaflet invasive complications, were exploratory, were not adjusted for multiple comparisons, and were not interpreted according to statistical significance alone. For the comparison of infection-related reconstruction within the invasive subgroup, an unadjusted prevalence ratio (PR) comparing BAV with TAV and its 95% confidence interval was calculated using the log-Wald method.

Long-term survival was estimated by the Kaplan–Meier method and compared with the log-rank test. Median follow-up was estimated by the reverse Kaplan–Meier method [18]. Cox proportional hazards regression evaluated the association between BAV morphology and all-cause mortality, adjusting for age, sex, and the non-age Charlson comorbidity score. Standardized survival curves were generated from the Cox model by averaging patient-level predictions under BAV and TAV morphology. Given 46 deaths among 317 patients, the Cox model was restricted to these prespecified covariates. The proportional hazards assumption was assessed with Schoenfeld residuals. Tests were 2-sided, with *p* < 0.05 considered statistically significant. Analyses were performed using R version 4.6.0 (R Foundation for Statistical Computing).

## 3. Results

### 3.1. Study Population and Baseline Characteristics

Of 371 consecutive adults undergoing surgery for AV-IE, 43 with prosthetic valve IE were excluded. A further 11 were excluded because extensive leaflet destruction prevented reliable intraoperative classification of valve morphology. The final cohort comprised 317 patients, including 113 with BAV (35.6%) and 204 with TAV (64.4%) (Figure 1). Using operative classification as the reference, preoperative TTE correctly identified BAV in 92/113 patients (81.4%) and TAV in 195/204 (95.6%). TTE morphology was indeterminate in four included patients (1.3%) but could be determined intraoperatively.

Patients with BAV were younger than those with TAV (mean age, 43.3 vs. 50.9 years; SMD, 0.56) and more frequently male (89.4% vs. 77.0%; SMD, 0.34). They had a lower non-age Charlson comorbidity score (median, 1 vs. 2; SMD, 0.40), lower European System for Cardiac Operative Risk Evaluation II (EuroSCORE II) scores (median, 2.5% vs. 3.5%; SMD, 0.20), and less previous cardiac surgery (0% vs. 9.8%; SMD, 0.47). Congestive heart failure and New York Heart Association class III or IV symptoms were also less frequent in the BAV group (Table 1). Concomitant congenital heart disease was documented in 34 patients with TAV (16.7%) and 7 with BAV (6.2%; *p* = 0.008). Notably, all 11 patients with concomitant pulmonary valve IE in the TAV group had congenital structural cardiac abnormalities, including ventricular septal defects in 8 patients, patent ductus arteriosus in 2 patients, and previously repaired tetralogy of Fallot in 1 patient. The proportions undergoing surgery <24 h, 24 h to 5 days, and >5 days after IE diagnosis were 7.1%, 10.6%, and 82.3% in the BAV group and 9.3%, 7.4%, and 83.3% in the TAV group, respectively (Table 1). Preoperative hemoglobin and estimated glomerular filtration rate were higher in BAV, whereas other laboratory findings were broadly similar (Supplementary Table S1). Streptococci were the most frequently identified microorganisms in both groups (49.6% in BAV and 50.0% in TAV). The distributions of major microbiological categories appeared broadly similar between groups (Supplementary Table S2).

### 3.2. Operative Anatomical Findings

Before adjudication, inter-reviewer agreement was 100% for valve morphology, 99.1% for non-leaflet invasive complications, and 100% for infection-related reconstruction. The corresponding Cohen’s κ values were 1.00, 0.98, and 1.00, respectively.

Aortic valve vegetations were identified in 254 patients (80.1%): 86.7% of patients with BAV and 76.5% with TAV. Vegetation size, leaflet perforation, leaflet abscess, and leaflet tissue necrosis or loss were descriptively similar between groups. Vegetations on non-leaflet structures were documented in 30.1% of BAV and 14.7% of TAV cases (Table 2).

Any non-leaflet invasive complication was identified in 88 patients (27.8%): 50 of 113 with BAV (44.2%) and 38 of 204 with TAV (18.6%; *p* < 0.001). Abscess involving non-leaflet structures was more frequent in BAV than in TAV (43.4% vs. 16.7%; *p* < 0.001). Pseudoaneurysm and intracardiac fistula were uncommon and did not show clear group differences.

In the full cohort, aortic annular and LVOT involvement were observed more frequently in patients with BAV (Table 2). Other site-specific findings and multiregional involvement are summarized in Table 2. Because sites overlapped and the comparisons were exploratory and unadjusted for multiple testing, these findings were not interpreted as evidence of a morphology-specific route of spread. Multiregional involvement was documented in 23.9% of BAV and 11.3% of TAV cases.

Among the 88 patients with established non-leaflet invasion, site-specific comparisons were exploratory and did not establish a distinct morphology-specific anatomical distribution. Multiregional involvement was present in 54.0% of BAV and 60.5% of TAV cases (*p* = 0.540); site-specific findings are reported descriptively in Supplementary Table S3.

### 3.3. Operative Strategy and Infection-Related Reconstruction

Prosthetic aortic valve replacement (AVR) was performed in 302 patients (95.3%), including 107 of 113 patients with BAV (94.7%) and 195 of 204 patients with TAV (95.6%). Among patients undergoing prosthetic AVR, mechanical prostheses were used in 85.0% of BAV and 73.8% of TAV cases; the corresponding proportions for bioprostheses were 15.0% and 26.2% (*p* = 0.030 for the comparison of prosthesis type). Concomitant mitral, tricuspid, and pulmonary valve procedures were more frequent in TAV. Median cardiopulmonary bypass and aortic cross-clamp times were 133 and 97 min in BAV and 163 and 117 min in TAV, respectively (Table 3).

Any infection-related reconstruction was performed in 67 patients (21.1%), including 36 with BAV (31.9%) and 31 with TAV (15.2%). Annular reconstruction was performed in 17.7% of BAV and 6.9% of TAV cases; the other individual reconstructive procedures were uncommon and are reported descriptively in Table 3. Aortic root replacement was performed in 13 patients, including 1 infection-related Bentall procedure with a bioprosthetic valved conduit and 12 Bentall procedures for root dilatation (8 mechanical and 4 bioprosthetic valved conduits).

In exploratory analyses among patients with a non-leaflet invasive complication, 67 of 88 (76.1%) underwent infection-related reconstruction: 36 of 50 with BAV (72.0%) and 31 of 38 with TAV (81.6%; unadjusted PR, 0.88; 95% CI, 0.70–1.11; *p* = 0.296) (Supplementary Table S3). Reconstruction was performed in 45 of 50 patients with multiregional involvement (90.0%) and 22 of 38 with single-structure involvement (57.9%; *p* < 0.001) (Supplementary Table S5).

### 3.4. Primary and Secondary Analyses

After adjustment for age, sex, surgical year, and non-age Charlson comorbidity score, BAV morphology was associated with a greater prevalence of any non-leaflet invasive complication (aPR, 2.08; 95% CI, 1.41–3.06; *p* < 0.001). The adjusted absolute BAV-minus-TAV difference was 21.1 percentage points (95% CI, 9.6–32.6) (Table 4).

In a secondary descriptive model, BAV morphology was associated with a higher overall prevalence of infection-related reconstruction (aPR, 1.89; 95% CI, 1.18–3.01; *p* = 0.008), with an adjusted absolute difference of 14.0 percentage points (95% CI, 3.3–24.8). This estimate summarized the overall reconstructive consequence and was not interpreted as an effect independent of invasive anatomy.

After exclusion of concomitant mitral valve IE, the association with non-leaflet invasive complications persisted (aPR, 2.31; 95% CI, 1.35–3.94; *p* = 0.002). The secondary reconstruction estimate remained above 1 but was less precise and included the null (aPR, 1.77; 95% CI, 0.95–3.29; *p* = 0.07). Using abscess involving non-leaflet structures as the narrower anatomical outcome yielded an aPR of 2.38 (95% CI, 1.59–3.56; *p* < 0.001). After excluding 41 patients with concomitant congenital heart disease, the association between BAV morphology and non-leaflet invasive complications persisted (aPR, 1.89; 95% CI, 1.25–2.86; *p* = 0.002). The association with infection-related reconstruction also remained (aPR, 1.75; 95% CI, 1.07–2.86; *p* = 0.02) (Supplementary Table S4). Restricted cubic spline models produced materially similar BAV estimates. Tests for nonlinearity in surgical year were borderline; however, flexible modeling of surgical year produced nearly identical BAV estimates, indicating that the estimated associations were not materially sensitive to its functional form (Supplementary Table S6).

### 3.5. Early Postoperative Outcomes

In-hospital mortality occurred in 15 patients (4.7%) and 30-day mortality in 18 (5.7%). The observed proportions were 4.4% versus 4.9% for in-hospital mortality and 6.2% versus 5.4% for 30-day mortality in BAV and TAV, respectively (*p* = 1.00 and *p* = 0.80) (Table 5). In unadjusted comparisons, prolonged mechanical ventilation (18.6% vs. 31.4%; *p* = 0.02) and re-thoracotomy (1.8% vs. 8.3%; *p* = 0.02) were less frequent in BAV. Median intensive care unit stay (2 vs. 3 days; *p* < 0.001) and postoperative hospital stay (9 vs. 11 days; *p* = 0.008) were also shorter. Other postoperative complications did not show clear group differences, although several event counts were small.

### 3.6. Long-Term Outcomes

**Figure 2:**
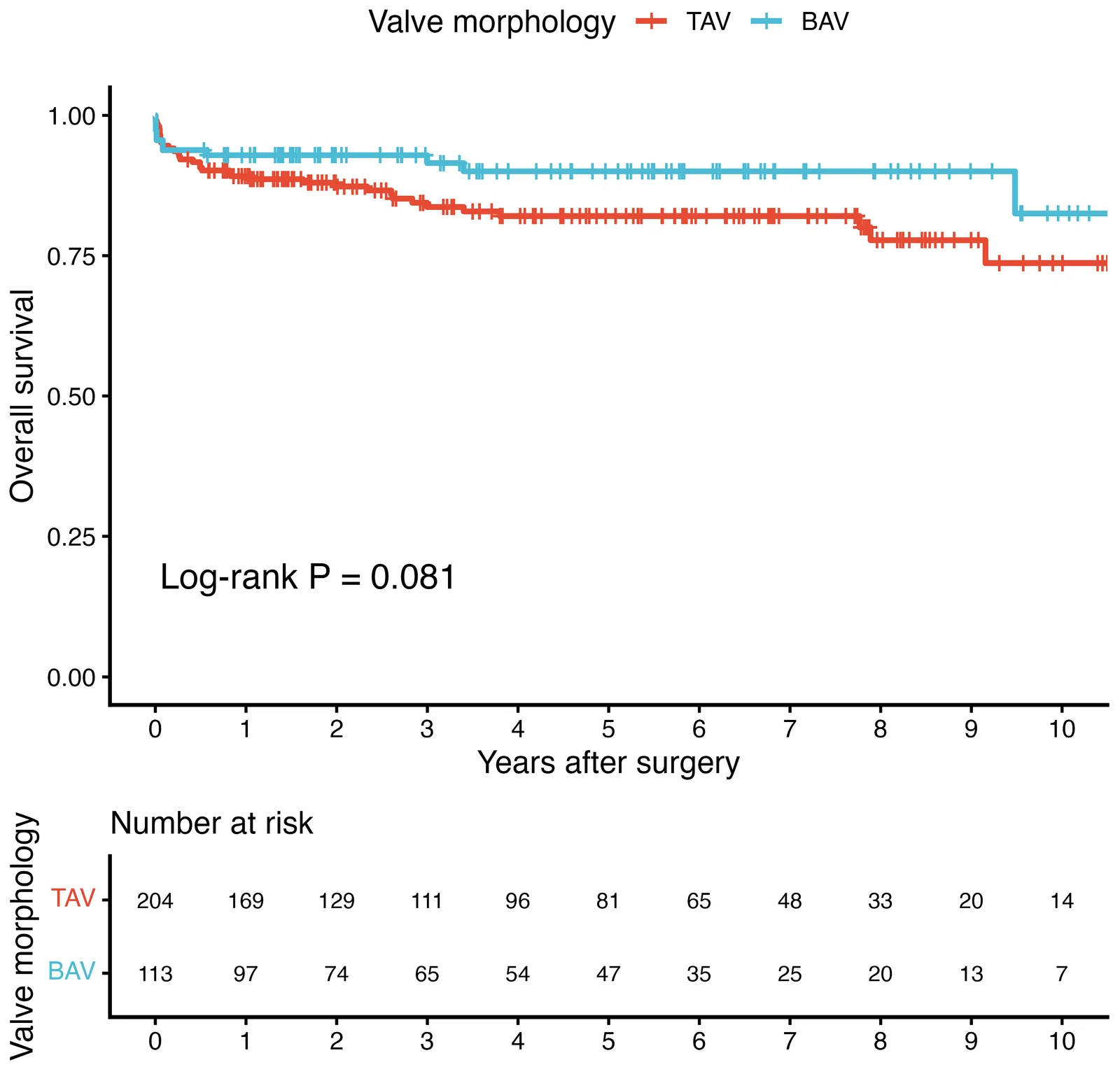
Kaplan–Meier Estimates of Long-Term Survival According to Native Aortic Valve Morphology. The log-rank *p* value was 0.081. BAV, bicuspid aortic valve; TAV, tricuspid aortic valve.

Three patients were lost to follow-up. Median follow-up was 4.68 years (95% CI, 4.02–5.43), during which 46 deaths occurred: 11 among patients with BAV (9.7%) and 35 among those with TAV (17.2%). Estimated 5-year survival was 90.0% (95% CI, 84.2–96.3%) in BAV and 82.1% (95% CI, 76.5–88.1%) in TAV; the log-rank *p* value was 0.081 (Figure 2; Supplementary Table S7).

**Figure 3:**
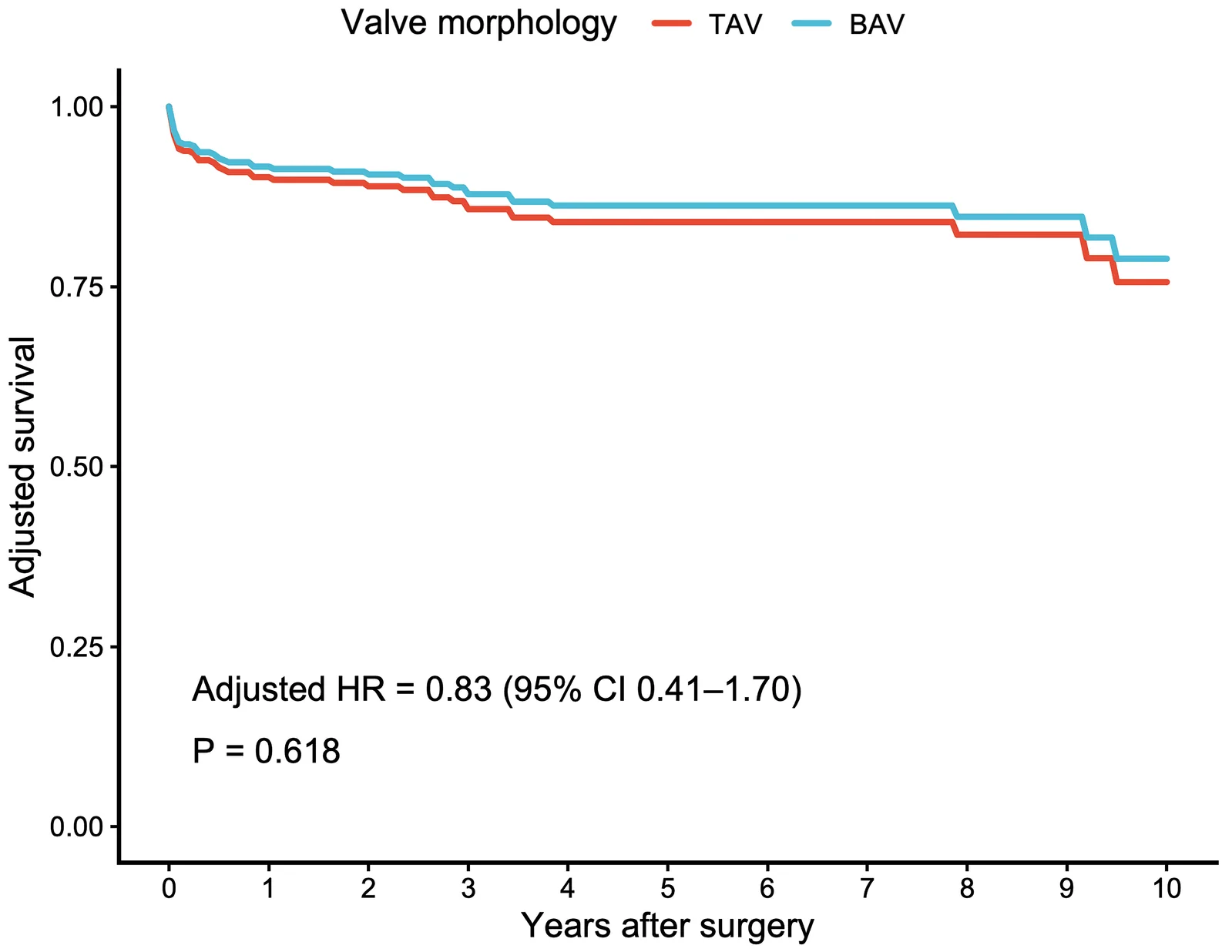
Adjusted Standardized Survival According to Native Aortic Valve Morphology. Model-based survival curves were derived from a Cox proportional hazards model adjusted for age, sex, and the non-age Charlson comorbidity score, with predicted survival probabilities averaged over the observed study cohort.

The unadjusted hazard ratio (HR) for BAV versus TAV was 0.55 (95% CI, 0.28–1.09; *p* = 0.087). After adjustment for age, sex, and the non-age Charlson comorbidity score, the adjusted HR was 0.83 (95% CI, 0.41–1.70; *p* = 0.618). The corresponding model-based standardized survival curves are shown in Figure 3. The adjusted estimate was imprecise and compatible with clinically important associations in either direction (Supplementary Table S7).

## 4. Discussion

In this surgical cohort of native AV-IE, BAV morphology was associated with a higher adjusted prevalence of non-leaflet invasive complications, predominantly abscess, and more frequent infection-related reconstruction. This study adds a standardized operative description of the non-leaflet structures involved and the reconstructions performed after debridement. Exploratory analyses among patients with established invasion did not establish consistent differences in anatomical involvement or the use of specific reconstructive procedures between BAV and TAV.

The association with invasive complications is consistent with previous reports [5,6,7,8,9,10,11,12,13,14]. Kahveci et al. reported periannular complications in 64% of BAV and 17% of TAV cases, whereas Bohbot et al. reported perivalvular complications in 34.1% and 16.5%, respectively [6,14]. In our cohort, non-leaflet invasive complications occurred in 44.2% and 18.6%, respectively (aPR, 2.08; 95% CI, 1.41–3.06). Differences in cohort selection, endpoint definitions, and assessment methods limit direct comparisons of absolute frequencies and effect estimates. More frequent annular and LVOT involvement in the full BAV cohort may reflect its higher prevalence of invasion. Anatomical sites overlapped, and exploratory comparisons were not adjusted for multiple testing. Site-specific and extent comparisons within the invasive subgroup were exploratory and insufficient for firm conclusions about morphology-related anatomical differences.

BAV patients were younger, more often male, and had fewer comorbidities, as reported previously [5,6,7,8,9,10,11,12,13,14]. The primary and secondary models adjusted for age, sex, surgical year, and non-age Charlson comorbidity score. These associations within a selected surgical cohort do not establish causal effects. Residual differences in referral, disease course, and other unmeasured factors may remain. Huntley et al. reported longer symptom-to-diagnosis intervals in BAV-associated IE than in TAV-associated IE, with diagnostic delay associated with IE-related adverse events [19]. The microbiological categories were descriptive and cannot exclude differences in strain-level virulence, prior antimicrobial exposure, or culture-negative infection.

Infection-related reconstruction characterizes the surgical management of invasive anatomy rather than an independent anatomical endpoint, and its frequency reflects both invasion prevalence and operative decision-making. Complete debridement and reconstruction tailored to the residual defect are central to surgical management [1,2,20,21,22,23]. Involvement of the mitral-aortic intervalvular fibrosa or adjacent structures may require patch reconstruction, a Commando or Hemi-Commando procedure, or other structural repair [24,25,26]. Among the 88 patients with established invasion, reconstruction was performed in 36 of 50 BAV cases (72.0%) and 31 of 38 TAV cases (81.6%; unadjusted PR, 0.88; 95% CI, 0.70–1.11; *p* = 0.296). This exploratory, unadjusted comparison was limited by sample size and potential selection bias. The confidence interval was compatible with both lower and higher reconstruction prevalence in BAV, and the findings do not establish equivalence between groups.

Sensitivity analyses supported the primary anatomical association. After exclusion of patients with concomitant mitral valve IE, BAV morphology remained associated with a higher prevalence of non-leaflet invasive complications, and the narrower outcome of abscess involving non-leaflet structures yielded a similar estimate. The secondary reconstruction estimate after excluding concomitant mitral valve IE remained above 1 but was imprecise and included the null (aPR, 1.77; 95% CI, 0.95–3.29). Spline models yielded similar morphology estimates, suggesting limited sensitivity to the modeled functional forms of age and surgical year.

More frequent concomitant valve procedures and longer operative times in TAV may have contributed to differences in postoperative recovery, although these unadjusted comparisons cannot establish mediation. Sparse early deaths preclude conclusions about equivalent perioperative safety. The unadjusted association between BAV and lower mortality was attenuated after adjustment for age, sex, and comorbidity. Previous studies have likewise compared survival in BAV and TAV populations with differing baseline characteristics [12,13,14]. The imprecise adjusted mortality estimate (HR, 0.83; 95% CI, 0.41–1.70) permits clinically important differences in either direction and provides secondary clinical context.

This retrospective tertiary-center study included only surgical patients, with potential bias from referral, survival to surgery, differential surgical selection, and exclusion of patients whose valve morphology was unclassifiable because of extensive leaflet destruction. Limited pre-IE records prevented reliable etiologic classification of pre-existing acquired aortic valve disease, leaving potential differences in underlying valve pathology unaccounted for. The findings do not estimate the overall burden of invasive AV-IE and may not generalize to nonsurgical patients.

## 5. Conclusions

Bicuspid morphology was associated with a higher prevalence of non-leaflet invasive complications, predominantly abscess. In the overall surgical cohort, aortic annular and LVOT involvement were more frequent in BAV. Infection-related reconstruction was also more frequent in BAV in the setting of more frequent invasion, but exploratory analyses did not demonstrate a distinct morphology-specific distribution or greater anatomical extent once invasion was established. These anatomical and reconstructive findings characterize operative anatomy and its reconstructive consequences without establishing a morphology-specific mechanism or operative strategy. In secondary survival analyses, no clear difference in adjusted long-term survival was identified between BAV and TAV.

## Figures and Tables

**Table 1:** Baseline and preoperative characteristics according to native aortic valve morphology.

Characteristics	Overall (N = 317)	TAV (N = 204)	BAV (N = 113)	*p* Value	SMD
Demographics and comorbidities					
Age, years	48.2 (14.0)	50.9 (13.6)	43.3 (13.6)	<0.001	0.56
Male sex	258 (81.4%)	157 (77.0%)	101 (89.4%)	0.007	0.34
Body mass index, kg/m^2^	22.8 (3.7)	23.0 (3.7)	22.2 (3.6)	0.06	0.22
Smoking	66 (20.8%)	40 (19.6%)	26 (23.0%)	0.47	0.08
Hypertension	82 (25.9%)	61 (29.9%)	21 (18.6%)	0.03	0.27
Diabetes mellitus	40 (12.6%)	28 (13.7%)	12 (10.6%)	0.48	0.10
Chronic obstructive pulmonary disease	6 (1.9%)	2 (1.0%)	4 (3.5%)	0.19	0.17
Coronary artery disease	40 (12.6%)	28 (13.7%)	12 (10.6%)	0.48	0.10
Peripheral vascular disease	36 (11.4%)	23 (11.3%)	13 (11.5%)	1.00	0.01
Dialysis	14 (4.4%)	11 (5.4%)	3 (2.7%)	0.39	0.14
Cardiac arrhythmias	13 (4.1%)	8 (3.9%)	5 (4.4%)	1.00	0.03
Autoimmune disease	18 (5.7%)	12 (5.9%)	6 (5.3%)	1.00	0.02
Cancer	8 (2.5%)	7 (3.4%)	1 (0.9%)	0.27	0.18
Previous cardiac surgery	20 (6.3%)	20 (9.8%)	0 (0.0%)	<0.001	0.47
Concomitant congenital heart disease	41 (12.9%)	34 (16.7%)	7 (6.2%)	0.008	0.33
Previous infective endocarditis	6 (1.9%)	6 (2.9%)	0 (0.0%)	0.09	0.25
Preoperative risk score					
Non-age Charlson comorbidity index	2 (1–3)	2 (1–4)	1 (1–3)	0.002	0.40
EuroSCORE II, %	3.1 (1.7–6.9)	3.5 (2.1–8.5)	2.5 (1.5–4.5)	<0.001	0.20
Echocardiography					
LVEF, %	62.8 (8.6)	62.4 (8.5)	63.7 (8.7)	0.20	0.15
LVEF < 50%	23 (7.3%)	15 (7.4%)	8 (7.1%)	1.00	0.01
Systolic pulmonary artery pressure > 55 mmHg	55 (17.4%)	39 (19.1%)	16 (14.2%)	0.28	0.13
Embolic events					
Overall embolism	134 (42.3%)	85 (41.7%)	49 (43.4%)	0.81	0.03
Cerebrovascular	84 (26.5%)	52 (25.5%)	32 (28.3%)	0.60	0.06
Lung	8 (2.5%)	8 (3.9%)	0 (0.0%)	0.05	0.29
Spleen	25 (7.9%)	18 (8.8%)	7 (6.2%)	0.52	0.10
IE Characteristics					
Mitral valve IE	105 (33.1%)	69 (33.8%)	36 (31.9%)	0.80	0.04
Tricuspid valve IE	21 (6.6%)	18 (8.8%)	3 (2.7%)	0.04	0.27
Pulmonary valve IE	11 (3.5%)	11 (5.4%)	0 (0.0%)	0.009	0.34
Healthcare-associated	14 (4.4%)	13 (6.4%)	1 (0.9%)	0.02	0.30
Injection drug use-associated	1 (0.3%)	1 (0.5%)	0 (0.0%)	1.00	0.10
Preoperative infection and clinical status					
NYHA class III/IV	124 (39.1%)	89 (43.6%)	35 (31.0%)	0.03	0.26
Sustained fever	31 (9.8%)	23 (11.3%)	8 (7.1%)	0.32	0.15
Congestive heart failure	119 (37.5%)	87 (42.6%)	32 (28.3%)	0.02	0.30
Pneumonia	20 (6.3%)	17 (8.3%)	3 (2.7%)	0.05	0.25
Preoperative antibiotic therapy < 7 days	18 (5.7%)	11 (5.4%)	7 (6.2%)	0.80	0.03
Positive last preoperative blood culture	64 (20.2%)	42 (20.6%)	22 (19.5%)	0.88	0.03
Critical preoperative state	40 (12.6%)	29 (14.2%)	11 (9.7%)	0.29	0.14
Interval from IE diagnosis to surgery					
<24 h	27 (8.5%)	19 (9.3%)	8 (7.1%)	0.54	0.08
24 h to 5 days	27 (8.5%)	15 (7.4%)	12 (10.6%)	0.40	0.11
>5 days	263 (83.0%)	170 (83.3%)	93 (82.3%)	0.88	0.03

Values are reported as *n* (%), median (IQR), or mean (standard deviation). SMDs are reported as absolute values. Abbreviations: BAV, bicuspid aortic valve; IE, infective endocarditis; EuroSCORE II, European System for Cardiac Operative Risk Evaluation II; IQR, interquartile range; LVEF, left ventricular ejection fraction; NYHA, New York Heart Association; SMD, standardized mean difference; TAV, tricuspid aortic valve.

**Table 2:** Operative anatomical findings according to native aortic valve morphology.

Variable	Overall (N = 317)	TAV (N = 204)	BAV (N = 113)	*p* Value
Aortic valve leaflet findings				
Vegetation	254 (80.1%)	156 (76.5%)	98 (86.7%)	0.028
Vegetation ≥ 10 mm	166 (52.4%)	106 (52.0%)	60 (53.1%)	0.846
Leaflet perforation	112 (35.3%)	73 (35.8%)	39 (34.5%)	0.821
Leaflet abscess	7 (2.2%)	4 (2.0%)	3 (2.7%)	0.703
Leaflet tissue necrosis/loss	47 (14.8%)	30 (14.7%)	17 (15.0%)	0.935
Vegetation on non-leaflet structures	64 (20.2%)	30 (14.7%)	34 (30.1%)	0.001
Non-leaflet invasive complications				
Any non-leaflet invasive complication	88 (27.8%)	38 (18.6%)	50 (44.2%)	<0.001
Abscess involving non-leaflet structures	83 (26.2%)	34 (16.7%)	49 (43.4%)	<0.001
Pseudoaneurysm	3 (0.9%)	2 (1.0%)	1 (0.9%)	1.000
Intracardiac fistula	11 (3.5%)	8 (3.9%)	3 (2.7%)	0.752
Anatomic sites of non-leaflet invasive involvement				
Aortic annulus	50 (15.8%)	23 (11.3%)	27 (23.9%)	0.003
Aortic root wall/sinus	41 (12.9%)	22 (10.8%)	19 (16.8%)	0.125
LVOT	55 (17.4%)	21 (10.3%)	34 (30.1%)	<0.001
Mitral-aortic intervalvular fibrosa	25 (7.9%)	11 (5.4%)	14 (12.4%)	0.027
Basal interventricular septum	27 (8.5%)	10 (4.9%)	17 (15.0%)	0.002
Anterior mitral leaflet ventricular surface	14 (4.4%)	3 (1.5%)	11 (9.7%)	0.001
Left ventricular free wall	3 (0.9%)	0 (0.0%)	3 (2.7%)	0.045
Left atrial roof	4 (1.3%)	3 (1.5%)	1 (0.9%)	1.000
Right atrium	7 (2.2%)	6 (2.9%)	1 (0.9%)	0.428
Right ventricle/RVOT	5 (1.6%)	2 (1.0%)	3 (2.7%)	0.352
Other adjacent structures	4 (1.3%)	1 (0.5%)	3 (2.7%)	0.131
Multiregional involvement	50 (15.8%)	23 (11.3%)	27 (23.9%)	0.003

Values are *n* (%). Anatomical sites and invasive components were not mutually exclusive. Other adjacent structures included the interatrial septum in 2 patients (1 with BAV and 1 with TAV), the aortopulmonary septum in 1 patient with BAV, and the pericardium in 1 patient with BAV. Annular and multiregional involvement partially overlapped: 35 patients had both, 15 had isolated annular involvement, and 15 had multiregional involvement without annular involvement. Abbreviations: BAV, bicuspid aortic valve; LVOT, left ventricular outflow tract; RVOT, right ventricular outflow tract; TAV, tricuspid aortic valve.

**Table 3:** Operative strategy and infection-related reconstruction according to native aortic valve morphology.

Variable	Overall (N = 317)	TAV (N = 204)	BAV (N = 113)	*p* Value
Aortic valve operative strategy				
Prosthetic AVR	302 (95.3%)	195 (95.6%)	107 (94.7%)	0.718
Mechanical prosthesis	235 (77.8%)	144 (73.8%)	91 (85.0%)	0.030
Bioprosthetic prosthesis	67 (22.2%)	51 (26.2%)	16 (15.0%)	
Manufacturer-labeled prosthesis size	23 (23–25)	23 (23–25)	23 (23–25)	0.530
Attempted AV repair converted to replacement	3 (0.9%)	3 (1.5%)	0 (0.0%)	0.555
Native valve repair	11 (3.5%)	6 (2.9%)	5 (4.4%)	0.530
Vegetation debridement only	3 (0.9%)	3 (1.5%)	0 (0.0%)	0.555
Pericardial neocuspidization (Ozaki)	1 (0.3%)	0 (0.0%)	1 (0.9%)	0.356
Infection-related reconstruction				
Any infection-related reconstruction	67 (21.1%)	31 (15.2%)	36 (31.9%)	<0.001
Annular reconstruction	34 (10.7%)	14 (6.9%)	20 (17.7%)	0.003
Aortic sinus patch reconstruction	27 (8.5%)	13 (6.4%)	14 (12.4%)	0.066
Infection-related root replacement	1 (0.3%)	1 (0.5%)	0 (0.0%)	1.000
Interventricular septal reconstruction	15 (4.7%)	7 (3.4%)	8 (7.1%)	0.143
Fistula repair	11 (3.5%)	8 (3.9%)	3 (2.7%)	0.752
Mitral-aortic intervalvular fibrosa reconstruction	19 (6.0%)	11 (5.4%)	8 (7.1%)	0.544
Localized patch reconstruction	1 (0.3%)	0 (0.0%)	1 (0.9%)	0.356
Commando procedure	13 (4.1%)	8 (3.9%)	5 (4.4%)	1.000
Hemi-Commando procedure	5 (1.6%)	3 (1.5%)	2 (1.8%)	1.000
Other adjacent cardiac structure reconstruction	8 (2.5%)	3 (1.5%)	5 (4.4%)	0.139
Concomitant procedures				
CABG	21 (6.6%)	17 (8.3%)	4 (3.5%)	0.100
Aortic root replacement for root dilatation	12 (3.8%)	7 (3.4%)	5 (4.4%)	0.761
Mitral valve repair	68 (21.5%)	45 (22.1%)	23 (20.4%)	0.723
Mitral valve replacement	63 (19.9%)	50 (24.5%)	13 (11.5%)	0.005
Tricuspid valve repair/replacement	53 (16.7%)	43 (21.1%)	10 (8.8%)	0.005
Pulmonary valve repair/replacement	11 (3.5%)	11 (5.4%)	0 (0.0%)	0.009
Operative times				
Cardiopulmonary bypass time, min	156 (109–189)	163 (114–200)	133 (101–179)	0.001
Aortic cross-clamp time, min	111 (74–143)	117 (77–149)	97 (69–131)	0.005

Values are *n* (%) or median (IQR). Percentages for mechanical and bioprosthetic prostheses are calculated among patients undergoing prosthetic AVR. Prosthesis size is reported in the same population. Patients with attempted AV repair converted to replacement are included in the prosthetic AVR counts. Abbreviations: AV, aortic valve; AVR, aortic valve replacement; BAV, bicuspid aortic valve; CABG, coronary artery bypass grafting; IQR, interquartile range; TAV, tricuspid aortic valve.

**Table 4:** Adjusted Associations of Bicuspid Aortic Valve Morphology With Non-Leaflet Invasive Complications and Infection-Related Reconstruction.

Outcome	TAV, *n*/N (%)	BAV, *n*/N (%)	aPR (95% CI)	Adjusted Absolute Difference, Percentage Points (95% CI)	Adjusted *p*
Any non-leaflet invasive complication	38/204 (18.6%)	50/113 (44.2%)	2.08 (1.41–3.06)	21.1 (9.6–32.6)	<0.001
Any infection-related reconstruction	31/204 (15.2%)	36/113 (31.9%)	1.89 (1.18–3.01)	14.0 (3.3–24.8)	0.008

Observed event rates are *n*/N (%). Any non-leaflet invasive complication was the primary outcome. Infection-related reconstruction was evaluated as a secondary operative outcome reflecting the operative management of invasive anatomy. Models included all 317 patients and were adjusted for age, sex, surgical year, and the non-age Charlson comorbidity score, with TAV as the reference. Adjusted absolute differences are marginally standardized BAV-minus-TAV differences in percentage points. Abbreviations: aPR, adjusted prevalence ratio; BAV, bicuspid aortic valve; CI, confidence interval; TAV, tricuspid aortic valve.

**Table 5:** Early postoperative outcomes according to native aortic valve morphology.

Outcome	Overall N = 317	TAV N = 204	BAV N = 113	*p* Value
Postoperative complications				
Neurological complication	9 (2.8%)	6 (2.9%)	3 (2.7%)	1.00
Ischemic stroke	5 (1.6%)	5 (2.5%)	0 (0.0%)	0.16
Hemorrhagic stroke	4 (1.3%)	1 (0.5%)	3 (2.7%)	0.13
Prolonged mechanical ventilation (>72 h)	85 (26.8%)	64 (31.4%)	21 (18.6%)	0.02
Postoperative pneumonia	39 (12.3%)	28 (13.7%)	11 (9.7%)	0.37
New-onset atrial fibrillation	49 (15.5%)	36 (17.6%)	13 (11.5%)	0.19
Atrioventricular block	9 (2.8%)	7 (3.4%)	2 (1.8%)	0.50
Ventricular arrhythmia	18 (5.7%)	12 (5.9%)	6 (5.3%)	1.00
Re-thoracotomy	19 (6.0%)	17 (8.3%)	2 (1.8%)	0.02
Low cardiac output syndrome	32 (10.1%)	24 (11.8%)	8 (7.1%)	0.24
Acute kidney injury	131 (41.3%)	89 (43.6%)	42 (37.2%)	0.29
Gastrointestinal bleeding	3 (0.9%)	3 (1.5%)	0 (0.0%)	0.56
Multiple organ dysfunction syndrome	11 (3.5%)	9 (4.4%)	2 (1.8%)	0.34
Sepsis	14 (4.4%)	11 (5.4%)	3 (2.7%)	0.39
Wound infection	1 (0.3%)	1 (0.5%)	0 (0.0%)	1.00
Mechanical and organ support				
IABP	6 (1.9%)	5 (2.5%)	1 (0.9%)	0.43
ECMO	7 (2.2%)	5 (2.5%)	2 (1.8%)	1.00
CRRT	37 (11.7%)	28 (13.7%)	9 (8.0%)	0.15
Postoperative recovery				
ICU stay, days	2 (1–5)	3 (2–6)	2 (1–3)	<0.001
Postoperative hospital stay, days	10 (7–15)	11 (8–16)	9 (7–13)	0.008
Early mortality				
In-hospital mortality	15 (4.7%)	10 (4.9%)	5 (4.4%)	1.00
30-day mortality	18 (5.7%)	11 (5.4%)	7 (6.2%)	0.80

Values are reported as *n* (%) or median (IQR). Re-thoracotomy was defined as unplanned reopening of the chest for postoperative bleeding, cardiac tamponade, hemodynamic deterioration, or another surgery-related complication. Abbreviations: BAV, bicuspid aortic valve; CRRT, continuous renal replacement therapy; ECMO, extracorporeal membrane oxygenation; IABP, intra-aortic balloon pump; ICU, intensive care unit; IQR, interquartile range; TAV, tricuspid aortic valve.

## Data Availability

The data underlying this study are not publicly available because they contain potentially identifiable patient information and are subject to institutional privacy and ethical restrictions. De-identified data may be available from the corresponding author upon reasonable request and subject to approval by the relevant institutional authorities.

## Supplementary Materials

The following supporting information can be downloaded at https://www.mdpi.com/article/10.3390/jcdd13100521/s1: Table S1: Preoperative Laboratory Findings According to Native Aortic Valve Morphology, Table S2: Microbiological Findings According to Native Aortic Valve Morphology, Table S3: Anatomic sites and infection-related reconstruction among patients with non-leaflet invasive complications, Table S4: Primary and sensitivity analyses of the associations of bicuspid aortic valve morphology with the primary and secondary outcomes, Table S5: Any infection-related reconstruction according to the extent of non-leaflet invasive involvement, Table S6: Assessment of the Functional Forms of Age and Surgical Year in the Primary and Secondary Models, Table S7: Long-Term Survival According to Native Aortic Valve Morphology.

## Abbreviations

The following abbreviations are used in this manuscript:

aPR	Adjusted prevalence ratio
AV	Aortic valve
AV-IE	Aortic valve infective endocarditis
AVR	Aortic valve replacement
BAV	Bicuspid aortic valve
CI	Confidence interval
IE	Infective endocarditis
MRSA	Methicillin-resistant *Staphylococcus aureus*
LVOT	Left ventricular outflow tract
SMD	Standardized mean difference
TAV	Tricuspid aortic valve
