# Supplementary material for: Invasive Complications and Infection-Related Reconstruction in Bicuspid Versus Tricuspid Native Aortic Valve Endocarditis: A 12-Year Surgical Cohort Study
Source: J Cardiovasc Dev Dis. 2026 Oct 16;13(10):521. doi: 10.3390/jcdd13100521 (PMC13705353; doi:10.3390/jcdd13100521)
Supplement: Supplementary file 1 [file jcdd-13-00521-s001.zip › Supplementary Table S1. Preoperative laboratory findings according to native aortic valve morphology.pdf]

Supplementary Table S1. Preoperative laboratory findings according to native aortic valve morphology

**Variable**	**Overall** N = 317	**TAV** N = 204	**BAV** N = 113	**P value**	**SMD**
White blood cell count, $\times 10^9$/L	7.6 [5.9, 10.3]	7.7 [5.9, 10.3]	7.4 [5.8, 10.6]	0.81	0.08
Hemoglobin, g/L	105 [89, 119]	102 [86, 118]	111 [97, 121]	0.004	0.32
Platelet count, $\times 10^9$/L	193 [143, 249]	191 [146, 248]	201 [141, 257]	0.61	0.03
Albumin, g/L	34 [30, 37]	34 [30, 37]	35 [30, 38]	0.13	0.17
eGFR, mL/min/1.73 m^2	101 [78, 114]	98 [73, 111]	106 [87, 119]	0.006	0.28
C-reactive protein, mg/L	33.4 [12.9, 63.0]	31.7 [12.7, 62.2]	35.0 [13.3, 68.1]	0.53	0.01
Procalcitonin, ng/mL	0.31 [0.07, 0.50]	0.37 [0.09, 0.50]	0.24 [0.07, 0.54]	0.77	0.17
NT-proBNP, pg/mL	1,663 [428, 6,266]	1,832 [463, 6,723]	1,320 [392, 3,964]	0.19	0.19

Values are median (IQR). SMDs are reported as absolute values and were calculated from the underlying means and SDs. Abbreviations: BAV, bicuspid aortic valve; eGFR, estimated glomerular filtration rate; NT-proBNP, N-terminal pro-B-type natriuretic peptide; SMD, standardized mean difference; TAV, tricuspid aortic valve.
