# Supplementary material for: Invasive Complications and Infection-Related Reconstruction in Bicuspid Versus Tricuspid Native Aortic Valve Endocarditis: A 12-Year Surgical Cohort Study
Source: J Cardiovasc Dev Dis. 2026 Oct 16;13(10):521. doi: 10.3390/jcdd13100521 (PMC13705353; doi:10.3390/jcdd13100521)
Supplement: Supplementary file 1 [file jcdd-13-00521-s001.zip › Supplementary Table S2. Microbiological findings according to native aortic valve morphology.pdf]

Supplementary Table S2. Microbiological findings according to native aortic valve morphology

Microbiological finding	Overall N = 317	TAV N = 204	BAV N = 113	P value	SMD
Culture-negative infective endocarditis	65 (20.5%)	38 (18.6%)	27 (23.9%)	0.31	0.13
Streptococci	158 (49.8%)	102 (50.0%)	56 (49.6%)	1.00	0.01
Staphylococci	39 (12.3%)	27 (13.2%)	12 (10.6%)	0.59	0.08
S. aureus	28 (8.8%)	19 (9.3%)	9 (8.0%)	0.84	0.05
MRSA	15 (4.7%)	11 (5.4%)	4 (3.5%)	0.59	0.09
Enterococci	16 (5.0%)	14 (6.9%)	2 (1.8%)	0.06	0.25
HACEK organisms	4 (1.3%)	2 (1.0%)	2 (1.8%)	0.62	0.07
Fungi	3 (0.9%)	3 (1.5%)	0 (0.0%)	0.56	0.17
Other microorganisms	33 (10.4%)	19 (9.3%)	14 (12.4%)	0.44	0.10

Values are *n* (%). SMDs are reported as absolute values. Major microbiological categories were not mutually exclusive: one patient with polymicrobial infective endocarditis caused by *Streptococcus* anginosus and *Staphylococcus* haemolyticus was counted in both the *Streptococci* and *Staphylococci* categories. *S. aureus* is a subset of *staphylococci*, and MRSA is a subset of *S. aureus*. Other microorganisms comprised causative pathogens not classified as *streptococci*, *staphylococci*, *enterococci,* HACEK organisms, or fungi, including non-HACEK Gram-negative bacteria and other Gram-positive bacteria. Culture-negative infective endocarditis was defined as the absence of an identified causative pathogen after all available microbiological investigations performed, including blood cultures and, when available, valve tissue culture, PCR/sequencing, and serological testing. Patients with pathogens identified by additional testing were classified according to the identified organism. Abbreviations: BAV, bicuspid aortic valve; HACEK, Haemophilus species, Aggregatibacter species, Cardiobacterium hominis, Eikenella corrodens, and Kingella species; MRSA, methicillin-resistant Staphylococcus aureus; SMD, standardized mean difference; TAV, tricuspid aortic valve.
