# Supplementary material for: Invasive Complications and Infection-Related Reconstruction in Bicuspid Versus Tricuspid Native Aortic Valve Endocarditis: A 12-Year Surgical Cohort Study
Source: J Cardiovasc Dev Dis. 2026 Oct 16;13(10):521. doi: 10.3390/jcdd13100521 (PMC13705353; doi:10.3390/jcdd13100521)
Supplement: Supplementary file 1 [file jcdd-13-00521-s001.zip › Supplementary Table S3. Invasive anatomy and infection-related reconstruction among patients with non-leaflet invasive complications.pdf]

Supplementary Table S3. Anatomic sites and infection-related reconstruction among patients with non-leaflet invasive complications

Variable	Overall (N = 88)	TAV (N = 38)	BAV (N = 50)	P value
Vegetation on non-leaflet structures	26 (29.5%)	5 (13.2%)	21 (42.0%)	0.003
Non-leaflet invasive complications				
Abscess involving non-leaflet structures	83 (94.3%)	34 (89.5%)	49 (98.0%)	0.161
Pseudoaneurysm	3 (3.4%)	2 (5.3%)	1 (2.0%)	0.576
Intracardiac fistula	11 (12.5%)	8 (21.1%)	3 (6.0%)	0.050
Anatomic sites of non-leaflet invasive involvement				
Aortic annulus	50 (56.8%)	23 (60.5%)	27 (54.0%)	0.540
Aortic root wall / sinus	41 (46.6%)	22 (57.9%)	19 (38.0%)	0.064
LVOT	55 (62.5%)	21 (55.3%)	34 (68.0%)	0.222
Mitral-aortic intervalvular fibrosa	25 (28.4%)	11 (28.9%)	14 (28.0%)	0.922
Basal interventricular septum	27 (30.7%)	10 (26.3%)	17 (34.0%)	0.439
Anterior mitral leaflet ventricular surface	14 (15.9%)	3 (7.9%)	11 (22.0%)	0.073
Left ventricular free wall	3 (3.4%)	0 (0.0%)	3 (6.0%)	0.255
Left atrial roof	4 (4.5%)	3 (7.9%)	1 (2.0%)	0.311
Right atrium	7 (8.0%)	6 (15.8%)	1 (2.0%)	0.039
Right ventricle / RVOT	5 (5.7%)	2 (5.3%)	3 (6.0%)	1.000
Other adjacent structures	4 (4.5%)	1 (2.6%)	3 (6.0%)	0.631
Multiregional involvement	50 (56.8%)	23 (60.5%)	27 (54.0%)	0.540
Infection-related reconstruction				
Any infection-related reconstruction	67 (76.1%)	31 (81.6%)	36 (72.0%)	0.296
Annular reconstruction	34 (38.6%)	14 (36.8%)	20 (40.0%)	0.763
Aortic sinus patch reconstruction	27 (30.7%)	13 (34.2%)	14 (28.0%)	0.531
Infection-related root replacement	1 (1.1%)	1 (2.6%)	0 (0.0%)	0.432
Interventricular septal reconstruction	15 (17.0%)	7 (18.4%)	8 (16.0%)	0.765
Fistula repair	11 (12.5%)	8 (21.1%)	3 (6.0%)	0.050
Mitral-aortic intervalvular fibrosa reconstruction	19 (21.6%)	11 (28.9%)	8 (16.0%)	0.144
Localized patch reconstruction	1 (1.1%)	0 (0.0%)	1 (2.0%)	1.000
Commando procedure	13 (14.8%)	8 (21.1%)	5 (10.0%)	0.148
Hemi-Commando procedure	5 (5.7%)	3 (7.9%)	2 (4.0%)	0.648
Other adjacent cardiac structure reconstruction	8 (9.1%)	3 (7.9%)	5 (10.0%)	1.000

Values are *n* (%). Anatomical sites and reconstructive procedures were not mutually exclusive. Other adjacent structures are detailed in Table 2. For any infection-related reconstruction within the invasive subgroup, the unadjusted prevalence ratio comparing BAV with TAV was 0.88 (95% CI, 0.70–1.11), calculated using the log-Wald method. Abbreviations: BAV, bicuspid aortic valve; LVOT, left ventricular outflow tract; RVOT, right ventricular outflow tract; TAV, tricuspid aortic valve.
