# Supplementary material for: Invasive Complications and Infection-Related Reconstruction in Bicuspid Versus Tricuspid Native Aortic Valve Endocarditis: A 12-Year Surgical Cohort Study
Source: J Cardiovasc Dev Dis. 2026 Oct 16;13(10):521. doi: 10.3390/jcdd13100521 (PMC13705353; doi:10.3390/jcdd13100521)
Supplement: Supplementary file 1 [file jcdd-13-00521-s001.zip › Supplementary Table S4. Primary and Sensitivity Analyses of the Associations of Bicuspid Aortic Valve Morphology With the Primary and Secondary Outcomes.pdf]

Supplementary Table S4. Primary and Sensitivity Analyses of the Associations of Bicuspid Aortic Valve Morphology With the Primary and Secondary Outcomes

Analysis	Outcome	TAV, events/N (%)	BAV, events/N (%)	Crude PR (95% CI)	Crude P	aPR (95% CI)	Adjusted P
A. Primary analysis: full cohort (N = 317)							
	Any non-leaflet invasive complication	38/204 (18.6%)	50/113 (44.2%)	2.38 (1.67–3.38)	<0.001	2.08 (1.41–3.06)	<0.001
	Any infection-related reconstruction	31/204 (15.2%)	36/113 (31.9%)	2.10 (1.38–3.20)	<0.001	1.89 (1.18–3.01)	0.008
B. Sensitivity analysis: excluding concomitant MV-IE (N = 212)							
	Any non-leaflet invasive complication	22/135 (16.3%)	33/77 (42.9%)	2.63 (1.66–4.17)	<0.001	2.31 (1.35–3.94)	0.002
	Any infection-related reconstruction	19/135 (14.1%)	23/77 (29.9%)	2.12 (1.24–3.64)	0.006	1.77 (0.95–3.29)	0.070
C. Sensitivity analysis: narrower anatomical endpoint (N = 317)							
	Abscess involving non-leaflet structures	34/204 (16.7%)	49/113 (43.4%)	2.60 (1.79–3.78)	<0.001	2.38 (1.59–3.56)	<0.001
D. Sensitivity analysis: excluding concomitant congenital heart disease (N = 276)							
	Any non-leaflet invasive complication	34/170 (20.0%)	46/106 (43.4%)	2.17 (1.50–3.14)	<0.001	1.89 (1.25–2.86)	0.002
	Any infection-related reconstruction	28/170 (16.5%)	34/106 (32.1%)	1.95 (1.26–3.02)	0.003	1.75 (1.07–2.86)	0.02

Event rates are events/N (%). Any non-leaflet invasive complication was the primary outcome. Infection-related reconstruction was evaluated as a secondary operative outcome reflecting the operative management of invasive anatomy. Abscess involving non-leaflet structures was a narrower sensitivity definition of the primary outcome. PRs and aPRs were estimated with TAV as the reference; adjusted models included age, sex, surgical year, and the non-age Charlson comorbidity score. Abbreviations: aPR, adjusted prevalence ratio; AV-IE, aortic valve infective endocarditis; BAV, bicuspid aortic valve; CI, confidence interval; MV-IE, mitral valve infective endocarditis; PR, prevalence ratio; TAV, tricuspid aortic valve.
