# Supplementary material for: Invasive Complications and Infection-Related Reconstruction in Bicuspid Versus Tricuspid Native Aortic Valve Endocarditis: A 12-Year Surgical Cohort Study
Source: J Cardiovasc Dev Dis. 2026 Oct 16;13(10):521. doi: 10.3390/jcdd13100521 (PMC13705353; doi:10.3390/jcdd13100521)
Supplement: Supplementary file 1 [file jcdd-13-00521-s001.zip › Supplementary Table S5. Any infection-related reconstruction according to the extent of non-leaflet invasive involvement.pdf]

Supplementary Table S5. Any infection-related reconstruction according to the extent of non-leaflet invasive involvement

Extent of non-leaflet invasive involvement	Overall	TAV	BAV
Single-structure involvement	22/38 (57.9%)	9/15 (60.0%)	13/23 (56.5%)
Multiregional involvement (≥2 involved structures)	45/50 (90.0%)	22/23 (95.7%)	23/27 (85.2%)
Overall cohort with non-leaflet invasive complications	67/88 (76.1%)	31/38 (81.6%)	36/50 (72.0%)
P value for <2 vs ≥2 involved structures	<0.001	0.010	0.031

Values are patients undergoing reconstruction/total patients in the stratum [*n*/N (%)]. P values are from two-sided Fisher exact tests comparing <2 versus ≥2 involved structures within each column; they are not interaction tests. Abbreviations: BAV, bicuspid aortic valve; TAV, tricuspid aortic valve.
