# Supplementary material for: Invasive Complications and Infection-Related Reconstruction in Bicuspid Versus Tricuspid Native Aortic Valve Endocarditis: A 12-Year Surgical Cohort Study
Source: J Cardiovasc Dev Dis. 2026 Oct 16;13(10):521. doi: 10.3390/jcdd13100521 (PMC13705353; doi:10.3390/jcdd13100521)
Supplement: Supplementary file 1 [file jcdd-13-00521-s001.zip › Supplementary Table S6. Assessment of the Functional Forms of Age and Surgical Year in the Primary and Secondary Models.pdf]

Supplementary Table S6. Assessment of the Functional Forms of Age and Surgical Year in the Primary and Secondary Models

Outcome	Covariate specification	Knot locations	BAV aPR (95% CI)	*P* for nonlinearity
**Panel A. Functional form of age**				
Any non-leaflet invasive complication	Linear (per 10-y increase)	—	2.08 (1.41–3.06)	—
Any non-leaflet invasive complication	RCS, 3 knots	P10/P50/P90: 27, 50, 65 y	2.07 (1.40–3.04)	0.643
Any non-leaflet invasive complication	RCS, 4 knots	P5/P35/P65/P95: 23, 43, 55, 70 y	2.07 (1.40–3.04)	0.906
Any infection-related reconstruction	Linear (per 10-y increase)	—	1.89 (1.18–3.01)	—
Any infection-related reconstruction	RCS, 3 knots	P10/P50/P90: 27, 50, 65 y	1.88 (1.18–2.98)	0.608
Any infection-related reconstruction	RCS, 4 knots	P5/P35/P65/P95: 23, 43, 55, 70 y	1.88 (1.18–2.98)	0.884
**Panel B. Functional form of surgical year**				
Any non-leaflet invasive complication	Linear (per 5-y increase)	—	2.08 (1.41-3.06)	—
Any non-leaflet invasive complication	RCS, 3 knots	P10/P50/P90: 2015, 2020, 2024	2.10 (1.43-3.08)	0.05
Any infection-related reconstruction	Linear (per 5-y increase)	—	1.89 (1.18-3.01)	
Any infection-related reconstruction	RCS, 3 knots	P10/P50/P90: 2015, 2020, 2024	1.91 (1.20-3.04)	0.053

Values are aPRs (95% CIs) for BAV versus TAV. All models included 317 patients and were adjusted for sex and the non-age Charlson comorbidity score, with age and surgical year parameterized as shown. Surgical year remained linear in Panel A, and age remained linear in Panel B. P for nonlinearity tests the joint nonlinear spline terms. TAV was the reference. Abbreviations: aPR, adjusted prevalence ratio; BAV, bicuspid aortic valve; CI, confidence interval; RCS, restricted cubic spline; TAV, tricuspid aortic valve.
