# Supplementary material for: Invasive Complications and Infection-Related Reconstruction in Bicuspid Versus Tricuspid Native Aortic Valve Endocarditis: A 12-Year Surgical Cohort Study
Source: J Cardiovasc Dev Dis. 2026 Oct 16;13(10):521. doi: 10.3390/jcdd13100521 (PMC13705353; doi:10.3390/jcdd13100521)
Supplement: Supplementary file 1 [file jcdd-13-00521-s001.zip › Supplementary Table S7. Long-term survival according to native aortic valve morphology.pdf]

Supplementary Table S7. Long-term survival according to native aortic valve morphology

Outcome	Overall<br>N = 317	TAV<br>N = 204	BAV<br>N = 113	HR (95% CI)	P value
**Follow-up**					
Median follow-up, years (95% CI)	4.68 (4.02–5.43)	4.78 (4.02–5.81)	4.58 (3.54–5.50)	—	—
Death during follow-up, n (%)	46 (14.5%)	35 (17.2%)	11 (9.7%)	—	—
**Kaplan–Meier estimated survival**					
1-year survival, % (95% CI)	90.5% (87.3–93.8)	89.2% (85.0–93.5)	92.9% (88.3–97.8)	—	—
5-year survival, % (95% CI)	84.9% (80.6–89.3)	82.1% (76.5–88.1)	90.0% (84.2–96.3)	—	—
Log-rank comparison	—	—	—	—	0.081
**Cox proportional hazards analysis**					
Unadjusted BAV vs TAV	—	Reference	—	0.55 (0.28–1.09)	0.087
Adjusted BAV vs TAV	—	Reference	—	0.83 (0.41–1.70)	0.618

Values are *n* (%) or Kaplan–Meier estimates (95% CIs), as appropriate. Cox analyses included 317 patients and 46 deaths; the adjusted model included age, sex, and the non-age Charlson comorbidity score, with TAV as the reference. Abbreviations: BAV, bicuspid aortic valve; CI, confidence interval; HR, hazard ratio; TAV, tricuspid aortic valve.
